# Afatinib increases sensitivity to radiation in non-small cell lung cancer cells with acquired EGFR T790M mutation

**DOI:** 10.18632/oncotarget.3332

**Published:** 2015-01-21

**Authors:** Shirong Zhang, Xiaoliang Zheng, Haixiu Huang, Kan Wu, Bing Wang, Xufeng Chen, Shenglin Ma

**Affiliations:** ^1^ Department of Radiation Oncology, Affiliated Hangzhou Hospital of Nanjing Medical University, Hangzhou, Zhejiang, China; ^2^ Centre of Molecular Medicine, Zhejiang Academy of Medical Sciences, Hangzhou, Zhejiang, China; ^3^ Department of Pathology and Laboratory Medicine, University of California at Los Angeles, Los Angeles, CA, USA

**Keywords:** afatinib, radiosensitization, NSCLC, EGFR, T790M

## Abstract

Afatinib is a second-generation of epidermal growth factor receptor (EGFR) tyrosine kinase inhibitor and has shown a significant clinical benefit in non-small cell lung cancer (NSCLC) patients with EGFR-activating mutations. However, the potential therapeutic effects of afatinib combining with other modalities, including ionizing radiation (IR), are not well understood. In this study, we developed a gefitinib-resistant cell subline (PC-9-GR) with a secondary EGFR mutation (T790M) from NSCLC PC-9 cells after chronic exposures to increasing doses of gefitinib. The presence of afatinib significantly increases the cell killing effect of radiation in PC-9-GR cells harboring acquired T790M, but not in H1975 cells with *de novo* T790M or in H460 cells that express wild-type EGFR. In PC-9-GR cells, afatinib remarkable blocks baseline of EGFR and ERK phosphorylations, and causes delay of IR-induced AKT phosphorylation. Afatinib treatment also leads to increased apoptosis and suppressed DNA damage repair in irradiated PC-9-GR cells, and enhanced tumor growth inhibition when combined with IR in PC-9-GR xenografts. Our findings suggest a potential therapeutic impact of afatinib as a radiation sensitizer in lung cancer cells harboring acquired T790M mutation, providing a rationale for a clinical trial with combination of afatinib and radiation in NSCLCs with EGFR T790M mutation.

## INTRODUCTION

Lung cancer is the leading cause of cancer-related mortality in the world [1]. Despite the advances in the diagnosis and treatment, the overall survival of this disease remains disappointing, and 5-year survival rate is approximately 15% [2]. Novel therapeutic strategies are thus needed for further improvement of clinical outcomes. In past decades, the identification of mutations of epidermal growth factor receptor (EGFR) that result in constitutive activation, and their association with significant responses to chemotherapeutic tyrosine kinase inhibitors (TKIs) have led to new paradigm of molecular targeting therapeutic strategy for patients with non-small cell lung cancer (NSCLC) [3]. However, acquired TKI resistance remains to be a major problem for therapeutic failure, which ultimately develops in most of the patients treated with reversible first-generation of TKI for 10 to 16 months, and almost 50% of cases are caused by acquired or de novo T790M mutation [4-6]. For these patients with re-progressed tumors with developed TKIs-resistance, treatments with second-line chemotherapeutic agents such as XL-647 [7], dasatinib [8], and neratinib [9] also failed to show obvious improvement of clinical outcomes.

Afatinib is a second-generation of EGFR-TKI, with highly selective inhibition on activations of EGFR, HER2, and HER4 [10, 11]. The irreversible, covalent binding of afatinib leads to longer suppression of receptor kinase activity when compared to first-generation of EGFR-TKIs such as gefitinib and erlotinib. Afatinib has been clinically evaluated in the LUX-Lung series of trials, and has been reported to increase progression-free survival in patients with EGFR-activating mutations as both first- and second/third-line therapies [12, 13]. It has also been reported to effectively decrease the cell proliferation rates of NSCLC cell lines expressing T790M mutation and showed therapeutic potential in xenograft of murine lung tumor with EGFR L858R/790M double mutations [10]. Of interest, the phase II trial LUX-Lung 4 demonstrated the modest but notable activity of afatinib in patients who developed acquired resistance to erlotinib, gefitinib or both [13]. These results indicate a potential of afatinib be one of the most promising therapeutic agents against NSCLC tumors with exon19 deletion, exon21 L858R and/or exon20 T790M mutations in EGFR gene.

In this study, we tested a hypothesis that afatinib may also act as a potential radiosensitizing agent in NSCLC cells with acquired gefitinib resistance. Our results showed that afatinib could significantly increase the clonogenic cell death in response to radiotherapy in established gefitinib-resistance human lung cancer cell PC-9-GR subline bearing EGFR 19Del/T790M double mutations *in vitro*, and enhance the tumor growth inhibition in xenografts model.

## RESULTS

### Development of gefitinib-resistant PC-9-GR cells

In this study, we first used stepwise exposure to increasing concentrations of gefitinib to establish gefitinib-resistant lung cancer cells. For this, PC-9 cells were first treated with 8 nM of gefitinib, a dose equivalent to IC30 in parental PC-9, and were then exposed to increased doses of gefitinib until 8.0μM.

**Figure 1:**
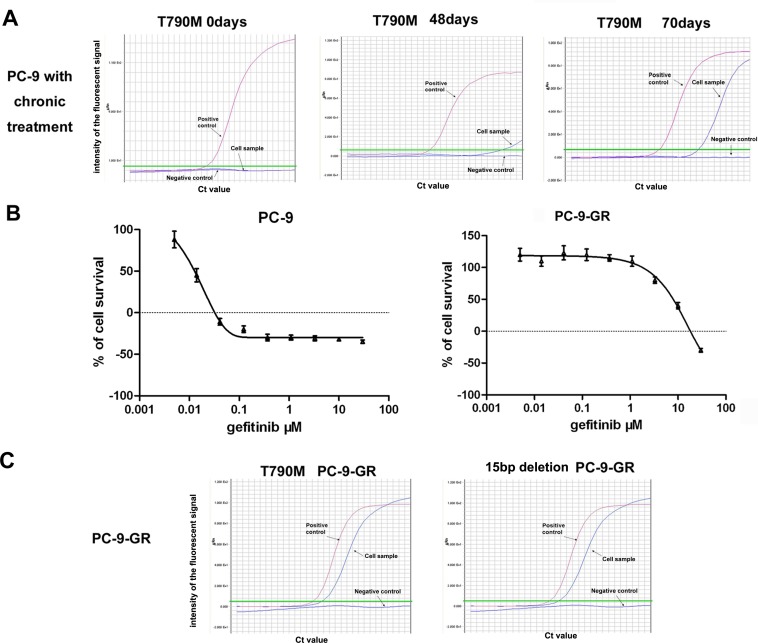
Development of gefitinib-resistant PC-9-GR cells (A). ARMS assay showing the emergency of EGFR T790M mutation in PC-9 cells exposed to chronic treatments of gefitinib. (B). Effects of gefitinib on cell growths of PC-9 and PC-9-GR cells. Cells were treated with different concentrations of gefitinib for 72 hours, and cell viabilities were determined by MTS assay. (C). Persistence of EGFR T790M in established PC-9-GR cells. ARMS assays were used to determine the T790M mutation and the 15 bp deletion of EGFR in cell samples with DNA extracted from PC-9-GR cells. ddH_2_O was used as negative control, and a DNA template with T790M mutation or 15bp deletion was included as positive control in these assays.

Previous studies have reported a secondary EGFR mutation at nucleotide 2369 in exon 20, which leads to a substitution of methionine for threonine at position 790 (T790M), in established gefitinib-resistance NSCLC cell lines, and in patients with acquired resistance after gefitinib treatment [14, 15]. We thus examined the potential emergency of EGFR T790M mutation in PC-9 cells during the chronic exposure to gefitinib. We detected EGFR T790M mutation with a assay of amplification refractory mutation system (ARMS) with sensitivity up to 0.1% in cells exposed to gefitinib for 48 days, which was further confirmed in cells treated with gefitinib for 70 days (Figure 1A).

Cells survived in the exposure to 8.0μM gefitinib were then subcloned, and direct sequencing for EGFR T790M mutation was performed in eleven selected clones. Of them, we found that six clones (near 50%) have detectable EGFR T790M mutation. A sub-clone with stable resistance to gefitinib and persistent EGFR T790M mutation was selected as established gefitinib-resistant subline (PC-9-GR).

With MTS assay, we found that the IC50 for gefitinib was 8.6 μM in PC-9-GR cells, which was about 680-fold higher than that in parental PC-9 cells (12.5 nM). No obvious morphologic change was observed in PC-9-GR cells comparing to PC-9 cells. We further noticed that maintenance in regular medium without gefitinib for >1 year did not change the decreased sensitivity to gefitinib of PC-9-GR cells (Figure 1B). However, we observed slight changes in cell cycle distribution in PC-9-GR cells. The cell growth analysis also showed that PC-9-GR has relatively slow growth kinetics, with longer cell doubling time (41.0 ± 3.1 vs 31.9 ± 3.3 hours), when compared to that of PC-9 cells (Supplementary Figure S1).

During the maintenance (>1 year) in drug-free medium, we detected the persistence of the EGFR T790M mutation, as well as the original 15 bp deletion of EGFR gene resulted from parental PC-9 cells, in PC-9-GR cells (Figure 1C and Supplementary Figure S2). No other mutation in the exons (exons 18-21) encoding the cytoplasmic region of the EGFR was detected in PC-9-GR cells.

### Afatinib radiosensitizes NSCLC cells with acquired gefitinib resistance

**Figure 2:**
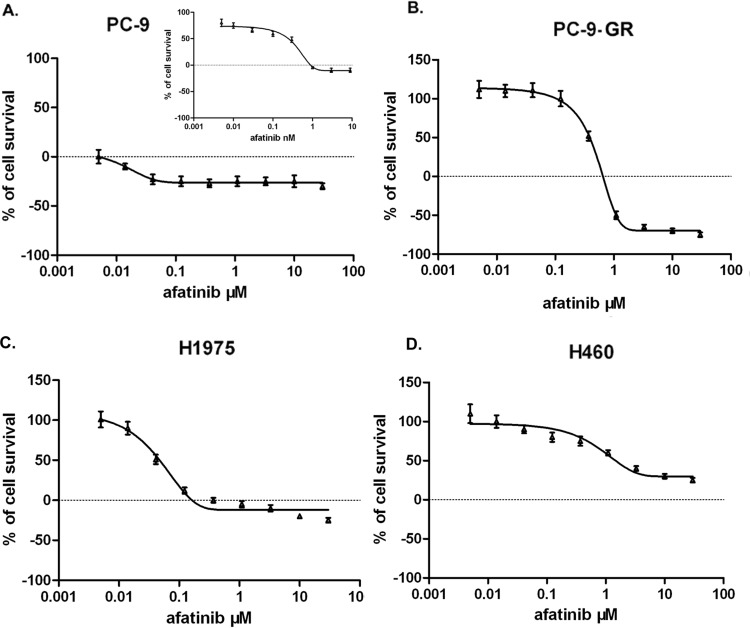
Effect of afatinib on cell growth in NSCLC PC-9 (A), PC-9-GR (B), H1975 (C) and H460 (D). Cells were treated with afatinib at indicated concentrations, and cell viabilities were determined using MTS assay. Data represent the average of three experiments. Error bars indicate standard deviation.

We next tested the sensitivities of PC-9 and PC-9-GR cells to afatinib treatment. For this, we determined the IC50 values of afatinib in these cells. We also evaluated IC50 values for afatinib in gefitinib-resistant human lung cancer H1975 cells carrying de novo T790M mutation, and in H460 cells which express wild-type of EGFR but carrying KRAS mutation which leads to gefitinib resistance. We found that IC50 values were 0.28 nM for PC-9, 350.0 nM for PC-9-GR, 38.4nM for H1975 and 2.3μM for H460 cells, respectively. These results suggest that NSCLC cells with T790M mutant EGFR are relatively sensitive to afatinib than cells carrying wild-type EGFR, which is consistent with the reports from previous study [16]. Of note, we found that IC50 value of PC-9-GR cells were significantly higher than that of parental PC-9 cells, indicating that development of acquired resistance to gefitinib, or to other first-generation of TKIs, may also cause reduced sensitivity to second-generation of TKIs in NSCLC cells that were initially sensitive to these agents (Figure 2).

**Figure 3:**
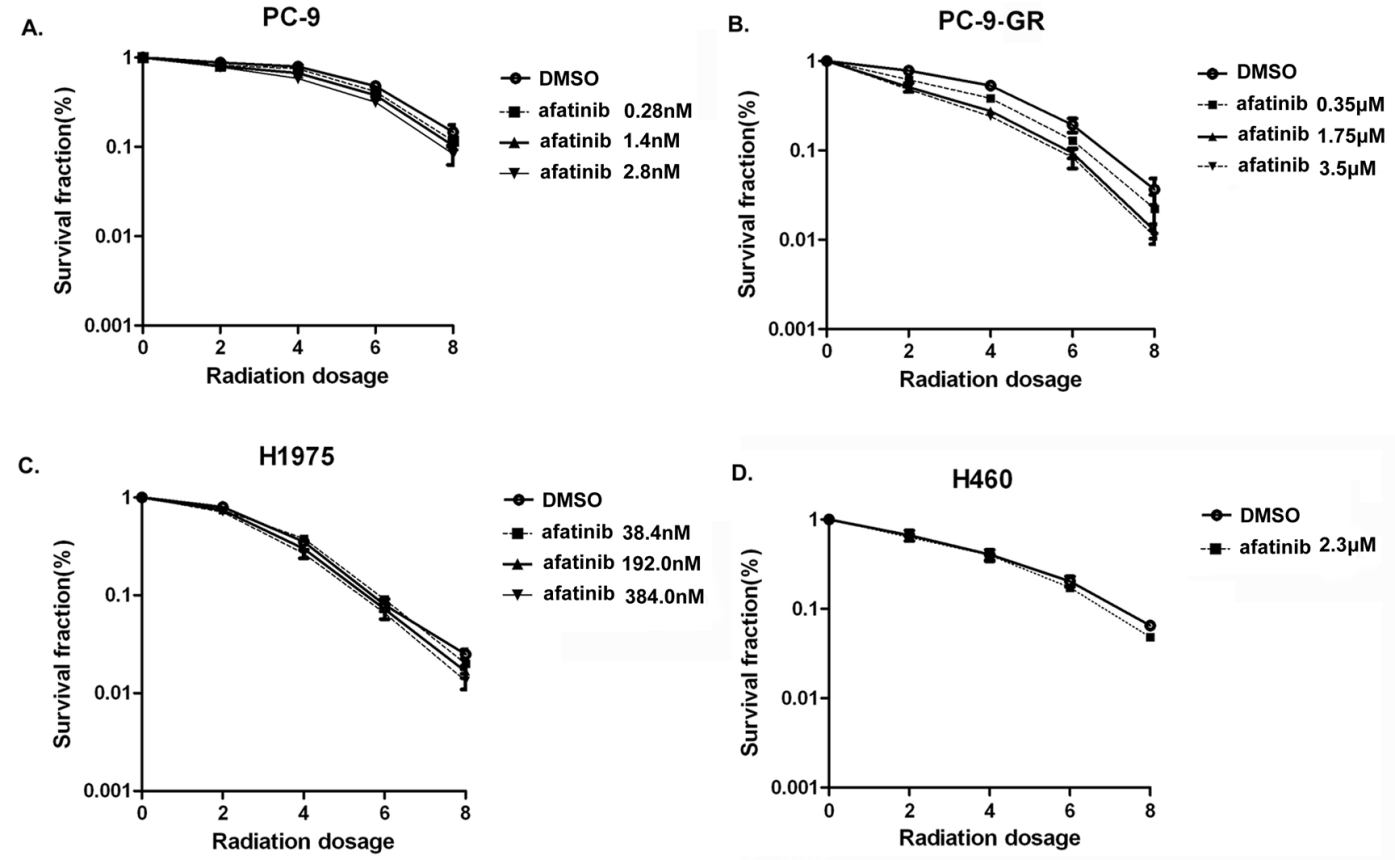
Effect of afatinib on cell clonogenic survival in irradiated lung cancer cells PC-9 (A), PC-9-GR (B), H1975 (C) and H460 (D) cells were pre-treated with afatinib for 2 hours and then irradiated with indicated doses. Clonogenic survival assays were performed as described in Materials and Methods. Data represent the average of three experiments. Error bars indicate standard deviation.

To evaluate potential radiosensitization effects of afatinib in these NSCLC cell lines, we determined the Sensitization Enhancement Ratio (SER) for clonogenic survival. In this study, cells were pretreated with corresponding IC50 values of afatinib and then irradiated. Our results showed that pretreatment with 350.0 nM of afatinib enhanced radiosensitivity of PC-9-GR cells (SER=1.22; *P*<0.0001). However, no obvious radiosensitization effects of afatinib were observed in H460 (SER=1.07), H1975 (SER=0.97), and PC-9 (SER=1.07) cells when exposed to corresponding IC50 values (Figure 3). Interestingly, we noticed that SER value for PC-9 cells was 1.22 (*P*<0.0001, Table 1) when cells were pretreated with afatinib at higher dose (2.8 nM, equivalent to 10×IC50), suggesting a potential radiosensitization effect of afatinib in a dose-dependent manner in PC-9 cells. Notably, higher SER values for PC-9-GR cells can also be reached when cells were pretreated with 1.75 μM (5×IC50, SER=1.41) or 3.5 μM (10×IC50, SER=1.50) of afatinib (*P*<0.0001) (Table 1). Interestingly, we did not observe radiosensitization effects of afatinib in H1975 cells even if cells were pretreated with higher concentration of afatinib (10×of corresponding IC50), although H1975 cells harbor EGFR T790M mutation and are sensitive to afatinib treatment. Because the concentrations of afatinib at doses of 11.5μM (5×IC50) and 23.0μM (10×IC50) are not clinical achievable, we did not test them for SERs in H460 cells. Nevertheless, these results suggest that afatinib may induce radiosensitizations in NSCLC cells carrying EGFR activating mutation and with acquired gefitinib-resistance.

**Table1:** Radiation sensitivity of NSCLC cell lines in the presence of afatinib

Afatinib Dose	IC50	5×IC50	10×IC50
SER for cell lines			
PC-9	1.07	1.13	1.22
PC-9-GR	1.22	1.41	1.50
H1975	0.97	1.05	1.10
H460	1.07	N/A	N/A

### Effects of afatinib on activations of EGFR, AKT and ERK proteins in NSCLC cells expressing T790M mutant EGFR in response to ionizing radiation treatment

**Figure 4:**
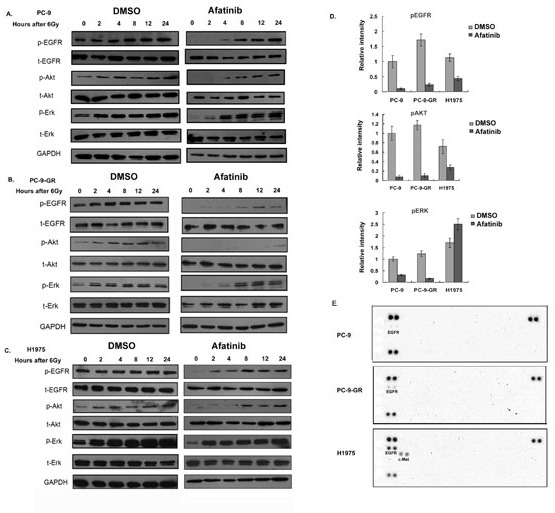
Effects of afatinib on protein phosphorylations (A-C). Western blot analysis. Cells were pretreated with afatinib for two hours followed by IR. Total cell lysates were collected from PC-9 (A), PC-9-GR (B) and H1975 (C) cells after indicated treatments, and analyzed for phosphorylations of EGFR, AKT and ERK proteins. Anti-GAPDH antibody was included as a loading control. (D). Quantitative analysis for the changes of protein phosphorylations. Densitometry for western blot signal for samples collected two hours post-treatments was conducted, and intensity for the targeted protein/modification was normalized to corresponding GAPDH. Data represent the average results from three independent experiments. (E). RTK array. Cell lysates were collected two hours after IR treatment or IR combined with afatinib, relative phosphorylation level of human receptor tyrosine kinases were determined with Phospho-RTK Array assay as described in Material and Methods.

It has been reported that receptor tyrosine kinases and AKT signaling are activated by irradiation in NSCLC cells [17-19]. To investigate whether treatment of afatinib can affect these activations, we treated cells with corresponding IC50 of afatinib combined with 6 Gy of ionizing radiation (IR). We found that the phosphorylation levels of EGFR, AKT and ERK increased after IR in PC-9, PC-9-GR and H1975 cells. Pretreatment with afatinib remarkable blocked basal level of the phosphorylations of EGFR and ERK proteins, and caused delays of IR-induced phosphorylation of AKT in these cells (Figure 4A-D and Supplementary Figure S3). However, we found that afatinib exposure did not cause changes of the basal phosphorylation levels for EGFR, AKT and ERK proteins in H460 cells (Supplementary Figure S3). The results from RTK array assays further showed that additional phosphorylation of c-Met was activated in H1975 cells, but not in PC-9 and PC-9-GR cells, after 2 hours with 6 Gy IR combined with afatinib (Figure 4E).

### Afatinib enhances IR-induced apoptosis and causes DNA damage repair delay in PC-9-GR cells through reduced DNA-pKcs expression

**Figure 5:**
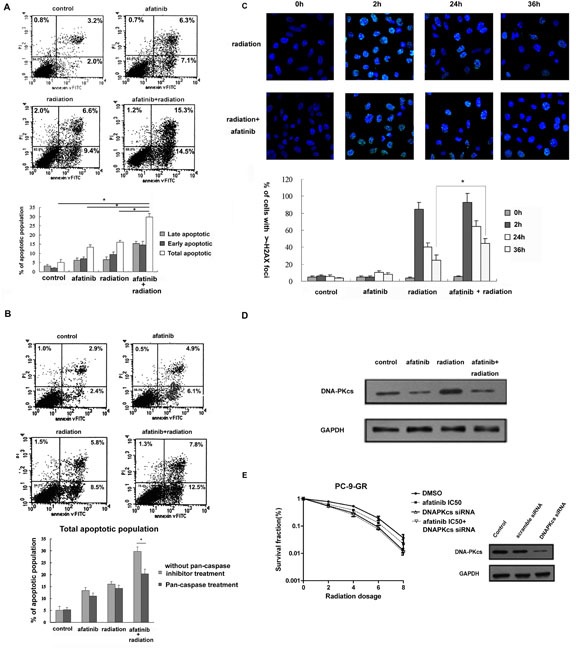
Effects of Afatinib on apoptotic response and DNA damage repair in irradiated PC-9-GR cells (A). Apoptosis analysis. Cells were treated with afatinib (350.0 nM), IR (6Gy) or afatinib for two hours followed by IR. Apoptosis was determined 36 hours later after IR with flow cytometry analysis. (B). Effect of pan-caspase inhibitor z-VAD-fmk on afatinib-enhanced apoptotic response in irradiated PC-9-GR cells. Cells were pretreated with 20 M z-VAD-fmk, followed by indicated treatments. Apoptosis was determined with flow cytometry analysis. Diagram showing the changes of early, late and total apoptosis in these experiments. (C). Immunofluorescence analysis. Immunofluorescence analyses were performed to examine the formation and changes of nuclear γ-H2AX foci. Representative images of nuclear γ-H2AX foci in PC-9-GR cells treated with IR or the combination are shown in top panel, and diagram showing the change in the cell fractions with positive γ-H2AX foci are present in bottom panel. Data represent the average of three experiments. Error bars indicate standard deviation. * indicates statistical significance. (D). Effect of afatinib on DNA-pKcs expression. Cell lysates were collected from PC-9-GR cells treated with afatinib, IR or the combination for twenty-four hours. Expression of DNA-pKcs protein was examined with western blot assay. Anti-GAPDH antibody was included as a loading control. (E). Clonogenic survival assay. PC-9-GR cells were transiently transfected with DNA-pKcs siRNA, or scramble-siRNA as control. Clonogenic survival assay was performed as described above. Western blot results showing the inhibitory effect of siRNAs on DNA-pKcs protein in cells collected 72 hours after transfections.

We next assessed the effects of afatinib on IR-induced apoptosis in PC-9-GR cells. As shown in Figure 5A, combination treatment of afatinib and IR induced significantly higher percentage (29.8%) of total apoptosis when compared to that in cells treated with afatinib or IR alone (13.4% and 16.0%, respectively). We also found that the presence of pan-caspase inhibitor z-VAD-fmk can effectively block the enhancement of apoptosis induced by afatinib in irradiated cells (Figure 5B).

IR-induced DNA double stand breaks (DSBs) is followed by γ-H2AX foci formation and recruitment of DNA-PK factors to sites of DSBs [20]. To test whether exposure to afatinib could affect DNA damage repair in irradiated cells, we determined the kinetic changes of γ-H2AX foci in PC-9-GR cells in response to IR treatment (Figure 5C). We found that cell fractions with discrete nuclear γ-H2AX foci dramatically increased (to 85%±8.2%) within 2 hour after 6 Gy of IR, and then dropped. By 36 hours post-IR, the percentage of cells with residual γ-H2A.X foci was 27%±5.1%. Exposure to afatinib did not cause obvious changes of baseline γ-H2AX foci formation. However, pretreatment with afatinib led to slightly increase of the formation of γ-H2AX foci (to 93%±11.3%) in cells after IR-exposure for 2 hours, and the number of cells with residual-H2A.X foci after 36 hours in these irradiated cells remained statistically higher at 45%±4.7%.

Previous study demonstrated that treatment with gefitinib could modulate the association of EGFR and DNA-pKcs leading to repressed function of DNA-pKcs [21]. We also found that treatment with afatinib blocked the increased expression of DNA-pKcs in PC-9-GR cells in response to IR (Figure 5D), indicating a potential involvement of NHEJ (non-homologous end-joining) repair pathway in cells treated with the combination of afatinib and IR. We thus tested the role of DNA-pKcs on afatinib-induced radiosensitization in PC-9-GR cells. We found that knocking-down of DNA-pKcs with siRNA transfection eliminated the radiosensitization effect of afatinib in PC-9-GR cells (Figure 5E). Thus, our results indicate a potential that afatinib exposure can cause persistence of lethal DNA damage with reduced NHEJ repair capability for IR-induced DNA DSBs, through modulation of DNA-pKcs expression.

### Combination of afatinib and radiation therapy inhibits tumor growth in PC-9-GR xenograft

**Figure 6:**
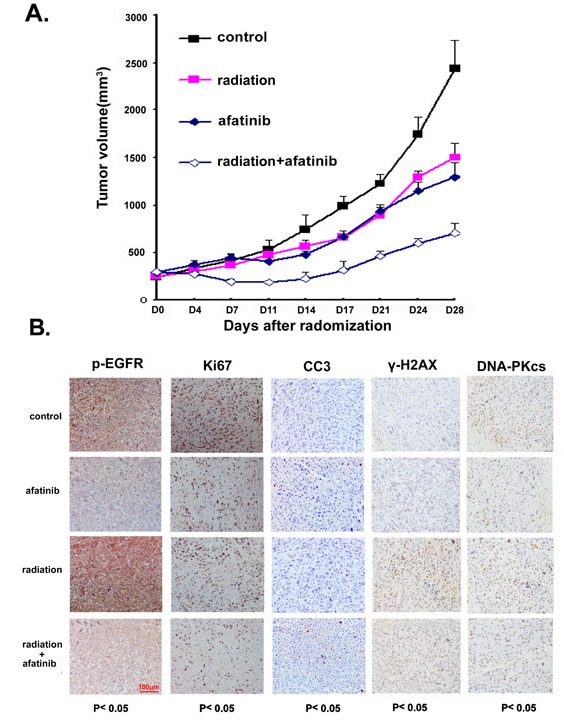
(A). Afatinib enhances tumor growth inhibition in response to IR treatment in PC-9-GR xenograft (A). Athymic nude mice bearing isogenic PC-9-GR xenograft tumors were treated with afatinib, IR or the combination. Tumor growth was measured as described in Materials and Methods. The growth curves represent the average values of 8 mice in each group. Error bars indicate standard deviation. (B). IHC staining. xenograft tumors tissues were collected after 14 days of indicated treatments. Immunostaining was performed to test the changes of EGFR phosphorylation, expressions of Ki67 and DNA-pKcs proteins, and presence of γ-H2A.X and apoptotic markers CC3. Quantified H-scores were determined for each group (n=3 animals/group). The scale bar represents 100μm and all images are to the same scale.

We further extended our study to an *in vivo* model. For this, PC-9-GR cells were inoculated into Nu/Nu mice to establish xenografts, and the effects of afatinib, IR or afatinib combined with IR on tumor growth were then assessed. Our results showed that treatment with single dose of IR (10 Gy) or daily oral treatment with afatinib (20mg/kg for 14 days) could inhibit *in vivo* PC-9-GR tumor growth with TGI of 38.4% and 46.9% respectively. However, we found that combination treatment of IR with afatinib caused significantly enhanced tumor growth inhibition with TGI of 71.1% (Figure 6A). In this experiment, we also measured the mice body weight to assess the tolerability of systemic therapies, and no obvious body weight changes were observed (Supplementary Figure S4), suggesting that treatment of IR combining with afatinib is well tolerable.

In a parallel experiment, we tested the changes of EGFR phosphorylation, expressions of molecular markers for cell proliferation (Ki-67) and apoptosis (cleavage of caspase 3), the presences of γ-H2AX and expression of DNA-pKcs in tumor tissues collected after treatments with immunohistochemistry analysis. Our data showed that afatinib suppressed phosphorylation of EGFR, even in cells where EGFR phosphorylation was enhanced by IR treatment; when compared to treatment with IR or afatinib alone, combined treatment of IR and afatinib increased the positive staining of cleaved caspase 3 (CC3) with statistical significance. We also noticed that, although treatment with IR or afatinib alone reduced staining of Ki67 in PC-9-GR tumors, combination treatment further decreased the level of positive staining for Ki67 in tumors tissues. Exposure to afatinib also suppressed IR-induced elevations of γ-H2AX foci formation and reduced DNA-pKcs expression in these tumor tissues (Figure 6B and Supplementary Table 1).

Taken together, our data suggest that afatinib can sensitize *in vivo* PC-9-GR tumor to radiation therapy.

## DISCUSSION

EGFR is a member of ErbB Family of receptors. The activation of the tyrosine kinase domain of EGFR activates EGFR pathways and results in the initiation of cancer proliferation, increased metastasis potential and neoangiogenesis. Thus, the mutated EGFR that lead to constitutive activation of EGFR signaling is oncogenic and is therefore attractive as a cancer therapeutic molecular target. Indeed, NSCLC patients with EGFR mutation can gain clinical benefit from EGFR TKIs as therapeutic agents. In addition, EGFR has been reported to play a role in the DNA damage response to radiation therapy [22, 23]. Following this, EGFR-TKIs have been reported to act as radiosensitizers in NSCLC and other cancers [24, 25].

Although the NSCLC tumors carrying mutated EGFR display significant responses (as high as 80%) to EGFR-TKIs, the cancer cells eventually become resistant to the treatment and median duration of response is about 10 to 16 months [6, 26]. Several mechanisms for the acquired resistance to these EGFR-TKIs have been identified, including a secondary EGFR mutation of T790M [14]. Previous studies demonstrated the emergence of T790M mutation in EGFR gene in established NSCLC cell lines with acquired gefitinib-resistance and in patients with prolonged treatment with EGFR-TKIs [27-29]. In this study, we also detected emergency of EGFR T790M mutation in NSCLC PC-9 cells exposed to chronic treatment of gefitinib, and in the established gefitinib-resistant PC-9-GR cells.

In NSCLC cells with initial EGFR-L858R mutation, the presence of secondary T790M mutation was found to affect the binding of gefitinib to mutated EGFR, with increased affinity of L858-T790M-EGFR to ATP when compared to L858 alone [30]. However, detailed mechanisms of how T790M affects the activation of mutated EGFR are still not clear. On the other hand, debate exists in regard to the selection or acquisition of this T790M mutation in EGFR gene in re-progressed cancer cells after treatment with EGFR-TKIs. Several studies suggested that T790M mutation was acquired only after exposure to EGFR-TKIs because pre-progression samples lacked T790M [31, 32]. Other reports, however, have indicated that T790M is not uncommon in tumors of TKI-naive patients with mutant EGFR, and clones with this alteration are selected for survival after treatment with EGFR-TKIs [9, 15, 33]. Nevertheless, this secondary T790M mutation negates the hypersensitivity of activated EGFR to EGFR-TKIs and generates high-grade resistance to achievable clinical doses of EGFR-TKIs.

Afatinib is the first irreversible ErbB Family Blocker, approved in the U.S, Europe, Taiwan and Mexico for use in patients with EGFR mutation-positive NSCLC. Unlike first generation of EGFR-TKIs, the irreversible binding of afatinib, which lead to combined inhibitions of ErbB family signaling, aims to provide a sustained, selective, covalent and complete ErbB family blockade. Afatinib has showed the preclinical activity in tumor models with EGFR-TKI resistant mutation T790M. A phase I/II trial (LUX-Lung 4) reported prolonged progression-free survival (PFS) for patients treated with afatinib when compared to the groups treated with either gefitinib or erlotinib [13]. Most interestingly, afatinib monotherapy or combination therapy has shown activity in patients with advanced NSCLC that had failed on previous treatment with gefitinib or erlotinib [10, 34]. Thus, afatinib may provide the potential benefits for NSCLC patients with mutant EGFR, when compared to first-generation EGFR-TKIs.

In this study, we investigated the effects of afatinib on mutant EGFR-bearing NSCLC cells with acquired gefitinib resistance in response to radiotherapy. Our results showed that the emergency of a secondary EGFR mutation, T790M, during the chronic exposure to gefitinib in PC-9 cells and in the consequently established gefitinib-resistant PC-9-GR subline cells. We also demonstrated in this study that afatinib could induce synergistic radiosensitization in PC-9-GR cells. The observed enhancements of tumor growth suppression and of irradiation-induced apoptosis in xenografts further supported the radiosensitization effect of afatinib in gefitinib-resistant PC-9-GR cells. To our best knowledge, this is the first study showing the combined effects of afatinib and radiotherapy in lung cancer cells with acquired EGFR-TKI resistance.

Radiation therapy is a key modality in the treatment of lung cancer. Although the molecular basis of radiation response is complex and multifactorial, the predominant mechanism by which therapeutic irradiation kills most tumor cells is through clonogenic death. DSBs are regarded as the specific lesions and main cause that initiates this lethal response and the repair of DSBs is then critical in determining radiosensitivity [35, 36]. Enhanced presences of γ-H2AX foci have been found in bladder cancer [37], human squamous cell carcinoma [38] and NSCLC cancer cells [39] treated with IR in the presence of TKIs, including afatinib. In PC-9-GR cells, pretreatment with afatinib also leads to increase and persistence of γ-H2AX foci in irradiated cells, and knocking-down of DNA-pKcs eliminates afatinib-induced radiosensitization. These results indicate that afatinib may affect the interaction of EGFR and DNA-pKcs [21], leading to NHEJ inhibition on DNA damage repair, in PC-9-GR cells in response to IR.

Previous study reported that combination treatment of irradiation and gefitinib had additive cell killing effects in activating EGFR mutations-bearing PC-9 and HCC827 cells [39]. In this study, we noticed that afatinib could sensitize PC-9 cells to IR when cells were exposed to relatively higher concentrations of afatinib. However, although H1975 cells carry EGFR mutation, and are relatively more sensitive to IR treatment alone when compared to PC-9-GR, no radiosensitization effects of afatinib were observed even if the cells were pretreated with afatinib at higher dose of 10×IC50. Of note, data from a previous study indicated that gefitinib could induce sensitivity in PC-9 cells, but not in H1975 cells [40], and combination of afatinib and c-Met inhibitor could enhance growth inhibition, apoptosis induction and inhibition of downstream signaling (including p-ERK [41, 42]) in H1975 cells [43-45]. Indeed, our results showed that c-Met was activated in H1975 cells after treatment of IR combining with afatinib. It is therefore possible that c-Met also plays important roles in cells in response to the combination treatment of IR and afatinib, and activation of c-MET may thus block afatinib-induced radiosensitization in H1975 cells. Further investigation is thus needed to test this hypothesis.

In conclusion, we demonstrated for the first time the *in vitro* and *in vivo* radiosensitization activities of afatinib in NSCLC cells with acquired gefitinib resistance. The data presented here suggest a potential clinic impact for use of afatinib as a part of radiotherapy regimen for NSCLC, and patients with acquired EGFR-TKI resistance may be further benefited from this therapeutic strategy.

## MATERIALS AND METHODS

### Cell culture and reagents

The human NSCLC PC-9, NCI-H1975 and NCI-H460 cell lines were obtained from the American Type Culture Collection (ATCC). These cells were maintained in an environment of 5% CO_2_ at 37°C in RPMI-1640 medium supplemented with 10% FBS. Afatinib (Cayman, San Diego, CA, USA) was dissolved in dimethyl sulfoxide (DMSO).

### Establishment of the gefitinib-resistant PC-9-GR subline cells from PC-9 cells

Human NSCLC cell line PC-9 was derived from an untreated Japanese patient with pulmonary adenocarcinoma that carried an in-frame deletion in EGFR exon 19 (delE746-A750) [46]. To establish gefitinib-resistant subline cells, PC-9 cells were first treated with 8 nM of gefitinib (Cayman, San Diego, CA, USA), and then were treated with increased concentrations of gefitinib in a stepwise manner during each passage and maintenance. The cells virtually survived in medium containing 8 μM of gefitinib (sixteen passages) were then seeded on 96-well plates for sub-cloning. After 21-28 days, clones from single cell were harvested and maintained in gefitinib-free medium. MTS assay were then performed to test gefitinib resistance. Direct sequencing was also applied for detection of EGFR T790M mutation in eleven selected clones. The subcloned cells were then maintained in gefitinib-free medium for at least one year, and their sensitivities to gefitinib and presence of T790M mutation were examined every 10 passages. Of them, a sub-clone with T790M mutation and stable resistance to gefitinib was further selected and termed as PC-9-GR for following experiments.

### Mutation analysis

Genomic DNA was extracted from the PC-9-GR cells using DNeasy Blood& Tissue Kit (Qiagen, Venlo, Netherland) per manufacturer's instruction. EGFR hotspot mutations were examined by ADx *EGFR* Mutations Detection Kit (Amoy Diagnostics, Xiamen, China). This kit uses the principle of Amplified Refractory Mutation System and covers the 29 *EGFR* mutation hotspots from exon 18 to 21. The assay was carried out according to the manufacturer's protocol with the ABI7900 (Applied Biosystems, USA) real-time PCR system. Direct DNA sequence analysis for Exon 18-21 of EGFR was also applied for detection of potential mutations in tyrosine kinase domain of EGFR gene.

### Cell proliferation analysis

Cell proliferation analysis was performed using MTS assay (tetrazolium-based CellTiter 96 Aqueous One Solution Proliferation assay, Promega, CA, USA) as per manufacturer's instruction. Briefly, cells were plated in 96-well plate (3000 cells/well). 24 hours later after plating, cells were treated with various concentration of afatinib, and cell viability was then determined 72 hours later.

### Clonogenic survival assay

Lung cancer cells in log phase were plated in six-well plates and pretreated with afatinib at indicated concentrations or DMSO as controls. Ionizing radiation (IR) was delivered 2 hours later. Irradiated cells were then maintained in RPMI-1640 medium containing 10% FBS for 14 to 20 days until colony counting. Colonies greater than 50 cells were counted as surviving colonies and the number of colonies was normalized to that observed in unirradiated controls. Mean inactivation doses were determined by the method of Fertil and colleagues [47], and the sensitizer enhancement ratio (SER) for afatinib treatment was calculated as the ratio of mean inactivation dose_control_/mean inactivation dose_afatinib -treated_.

For siRNA treatment, cells were transiently transfected with siRNA-control and siRNA-DNA-pKcs (Santa Cruz Biotech, CA). Forty-eight hours later, cells were pretreated with afatinib for 2 hours, and then be re-seeded and irradiated for clonogenic survival analysis.

### Immunofluorescence analysis

Cells were cultured on coverslips overnight and were then exposed to 6 Gy IR alone or IR combined with pretreatment of afatinib as described above. Cell samples were collected at indicated time intervals after IR, washed three times with ice-cold Ca^2+^/Mg^2+^-free phosphate-buffered saline (PBS), and fixed in 4% formaldehyde/PBS for 30 min. After permeabilization in 0.5% Triton X-100 in PBS for 10 minutes and blocking with 0.05% Tween + 5% BSA in PBS for 1 h at room temperature, cells were incubated with FITC-conjugated anti-phospho-Histone γ-H2AX (1:250, 9719s, signaling Technology, Danvers, MA, USA) for 2 h at room temperature, and then washed with PBS and mounted in Vectashield mounting medium containing diamidino-2-phenylindole (Vector Laboratories, CA). Images were acquired with LSM 510 confocal microscope (Zeiss, Germen) with 40×objective. For quantitative analysis, nuclei were analyzed by eye. At lease 150 cells from each experiment were selected at random and were counted to calculate the percentage of cells as “positive” for γ-H2AX if they displayed >5 discrete dots in nuclei. The results from at least 3 different experiments were averaged.

### Western blot analysis

Cells were collected and washed with cold PBS twice. Cells were then lysed in RIPA buffer containing protease and phosphatase inhibitors (Thermo Scientific) with mild sonication. 20μg of cell lysates were used for western blot analyses with antibodies against phospho-EGFR, EGFR, phospho-AKT, AKT, phospho-ERK, ERK, DNA-pKcs and GAPDH. All antibodies were purchase from cell signaling technology (CST, Danvers, MA, USA). To verify the changes of protein levels and protein modification, densitometry for western blot signal was conducted using Image Lab 5.0 software (BioRad Laboratories), and intensity for the targeted protein/modification was normalized to corresponding GAPDH.

### Phospho-receptor tyrosine kinase array assay

Human Phospho-RTK Array Kit (R&D Systems, ARY001B) was used to determine changes of phosphorylation level for forty-nine human receptor tyrosine kinases in PC-9, PC-9-GR and H1975 cells. Briefly, cell lysates were diluted and incubated with array. After binding with the extracellular domain of RTKs, unbound materials was washed away. A pan anti-phospho-tyrosine antibody conjugated to HRP was used to detect phosphorylated tyrosines on activated receptors by chemiluminescence.

### Assessment of apoptosis

Cells were pretreated with afatinib, or DMSO as control, for 2 hours followed with 6Gy IR. Cells were then collected 36 hours post-IR and stained with Annexin V-FITC (fluorescein isothiocyanate) as per manufacturer's instructions (Annexin V-FITC Apoptosis Detection Kit; BD Biosciences, USA). Briefly, 1×10^6^ cells were washed twice with cold PBS and stained with of Annexin V-FITC and PI in 1×binding buffer for 10 min at room temperature in the dark. The populations of apoptotic cells were determined using a Becton Dickinson FACScan cytofluorometer. Both early apoptotic (Annexin V-positive and PI-negative) and late apoptotic (Annexin V-positive and PI-positive) cells were included as total apoptosis.

For experiment with pan-caspase inhibitor, cells were pre-treated with the pan-caspase inhibitor z-VAD-fmk (20μM) for two hours, and then treated with afatinib, IR or the combination. Apoptotic populations of PC-9-GR were then determined by flow cytometry assay.

### Tumor growth assay

PC-9-GR cells (1×10^6^ in 0.2 mL HBSS 1× + 1% HSA) were inoculated subcutaneously into the right thigh of female Nu/Nu mice 4 to 6 weeks old (Charles River, Beijing, China). When the average tumor volume reached 200mm^3^, the mice were randomized into 4 groups to receive the following treatments: (a) methycellulose/Tween 80 as vehicle; (b) afatinib (20mg/kg/day) on day1-14; (c) ionizing radiation 10 Gy on day 5; and (d) afatinib + ionizing radiation 10 Gy. Tumors were measured twice weekly, and tumor volumes were determined from caliper measurements of tumor length (*L*) and width (*W*) according to the formula (*L* × *W*^2^)/2. Equation of [%TGI=(1- change of tumor volume in treatment group/change of tumor volume in control group)×100] was used to determine the percentage of tumor growth inhibition and antitumor efficacy.

### Immunohistochemistry analysis

PC-9-GR xenograft tumors were collected after 14 days of daily treatments as described above and fixed in 4% formalin. Antigen retrieval was conducted on FFPE tissues sections for 5 minutes with retrieval buffer (DAKO, Glostrup, Denmark) followed by washing in running water for 5 minutes. Tissue samples were then rinsed in TBS containing 1% Tween (TBST) and incubated with endogenous peroxidase blocker on a LabVision autostainer for 10 minutes. Slides were washed twice in TBST and incubated with primary antibodies (pEGFR, Abcam; CC3, DNA-pKcs, γ-H2AX, Cell Signaling Technology) for 60 minutes at room temperature and then washed twice in TBST. DAKO EnVision™+ System-HRP was used as second antibody for vitalization and staining was detected using diaminobenzidine (DAKO). For Ki67 immunohistochemical analysis, tumor sections were incubated with biotinylated primary antibody (DAKO) for 1 minute at room temperature and then washed twice with TBST. Following 15 minutes streptavidin-peroxidase treatment and washing with TBST, slides were counterstained with DAPI and visualized by chemiluminescence as described above. For analyses of baseline expression or modulation, IHC scoring of phospho-EGFR, γ-H2AX, and DNA-pKcs was conducted with using the following formula: scoring= 0×[% cells with no staining (0)]+1×[% cells staining faint to barely visible (1+)] +2×[% cells staining weak to moderately (2+)] +3×[% cells staining strongly (3+)]. This method combines positive intensity and percentage tumor cell staining and is determined by 2 separate pathologists using microscopy. Quantification of Ki67- and CC3-positive signals was conducted using the Ariol system (Genetix, San Jose, CA, USA).

### Statistical analysis

Data were presented as means ± SD. Student *t* test was used to determine the significance between groups. Significance was defined at the level of *P* <0.05.

## SUPPLEMENTARY MATERIAL TABLE AND FIGURES


