# Supplementary material for: Afatinib increases sensitivity to radiation in non-small cell lung cancer cells with acquired EGFR T790M mutation
Source: Oncotarget. 2015 Jan 21;6(8):5832–45. doi: 10.18632/oncotarget.3332 (PMC4467405; doi:10.18632/oncotarget.3332)
Supplement: Supplementary file 1 [file oncotarget-06-5832-s001.pdf]

Afatinib increases sensitivity to radiation in non-small cell lung cancer cells with acquired EGFR T790M mutation

Supplementary Material

Supplementary Table 1: IHC analysis of tumor sections showing modulation of pEGFR, Ki67, CC3, γ -H2AX and DNA-pKcs treated with afatinib and/or radiation.

IHC Marker		Treatment			
		control	afatinib	radiation	afatinib+ radiation
H score,(SD)	pEGFR	195,(14)	57,(11)	278,(10)	120,(17)
	γ-H2AX	7,(5)	43,(9)	154,(15)	76,(11)
	DNA-pKcs	81,(12)	40,(8)	102,(17)	51,(7)
IHC Positive staining %, (SD)	Ki67	70,(10)	32,(6)	43,(10)	19,(5)
	CC3	0.7,(0.08)	6,(2.3)	4.1,(1.6)	10.5,(2.8)

Athymic nude mice bearing isogenic PC-9-GR xenograft tumors were treated with afatinib and/or radiation. IHC stainings were performed in collected tumors tissues as described in Materials and Methods. Stained specimens were examined and "H" scores for pEGFR, γ-H2AX and DNA-pKcs were calculated as a composite measure of staining intensity and area. For Ki67and CC3, positive staining was assessed as "% area of staining" using Ariol image analysis. n=3 animals/group. SD – Standard Deviation.

Cell doubling time of PC-9 and PC-9-GR cells

Cell line	Cell doubling time (h)
PC-9	31.9 ± 3.3
PC-9-GR	41.0 ± 3.1

Cell growth curve

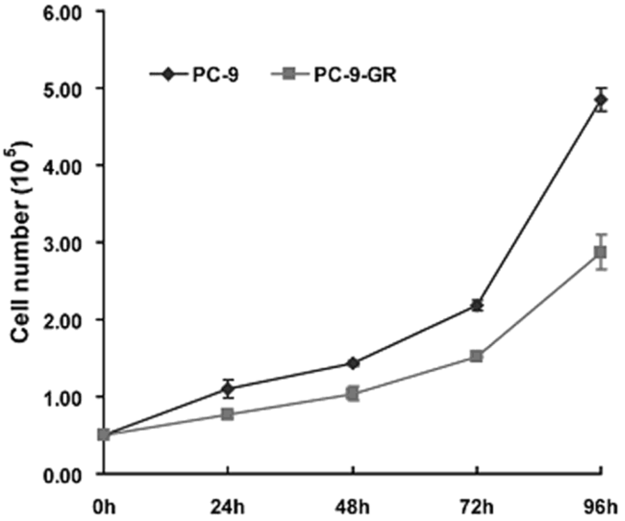

Cell cycle distribution of PC-9 and PC-9-GR cells

Cell line	Cell cycle (%)		
	G_0/G_1	S	G_2/M
PC-9	54.85 ± 1.57	35.70 ± 1.66	9.45 ± 0.55
PC-9-GR	49.03 ± 0.84	36.75 ± 3.02	14.22 ± 2.21

Supplementary figure S1: Characteristics of established PC-9-GR subline cells comparing to parental PC-9 cells. Cells were maintained in gefitinib-free medium, and then analyzed for: A. Cell doubling time; B. Cell growth curve; and C. Cell cycle distribution.

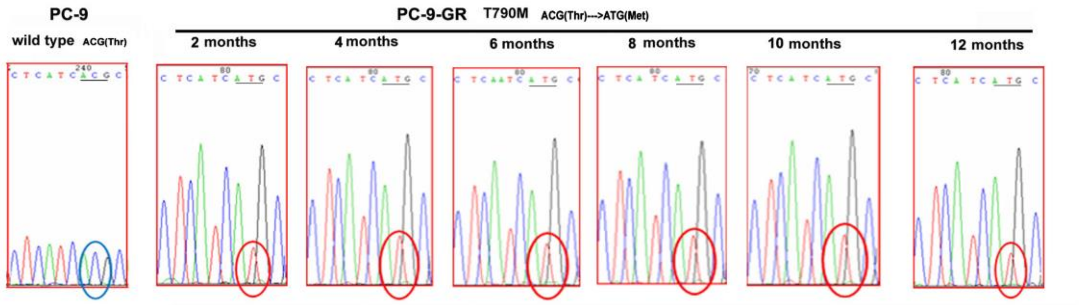

Supplementary figure S2: T790M mutation in PC-9-GR cells. The direct sequencing showed the widetype EGFR T790 in parental PC-9, and mutant EGFR T790M in established PC-9-GR during the maintenance in gefitinib-free medium.

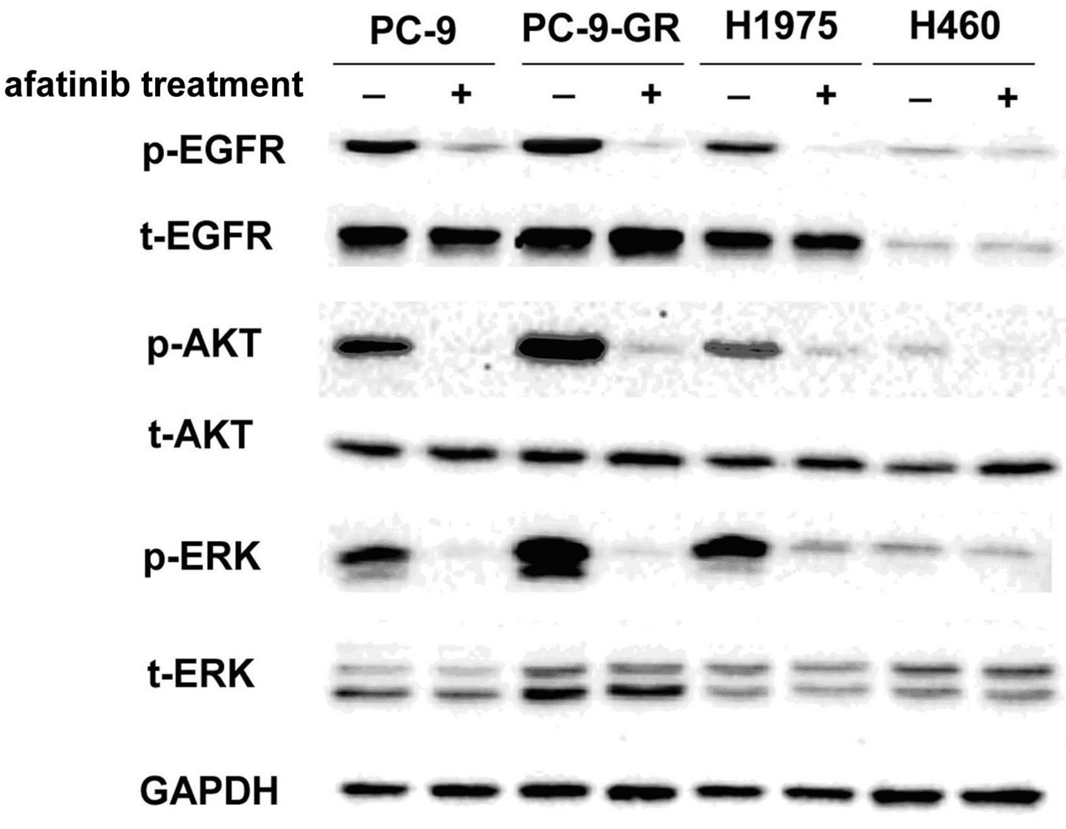

Supplementary figure S3: The effects of afatinib on protein phosphorylations in NSCLC cells. Cells were treated with afatinib at corresponding IC50 doses for two hours, and western blots were performed to test the changes of phosphorylation of EGFR, AKT and ERK proteins. GAPDH was included for protein equal loading.

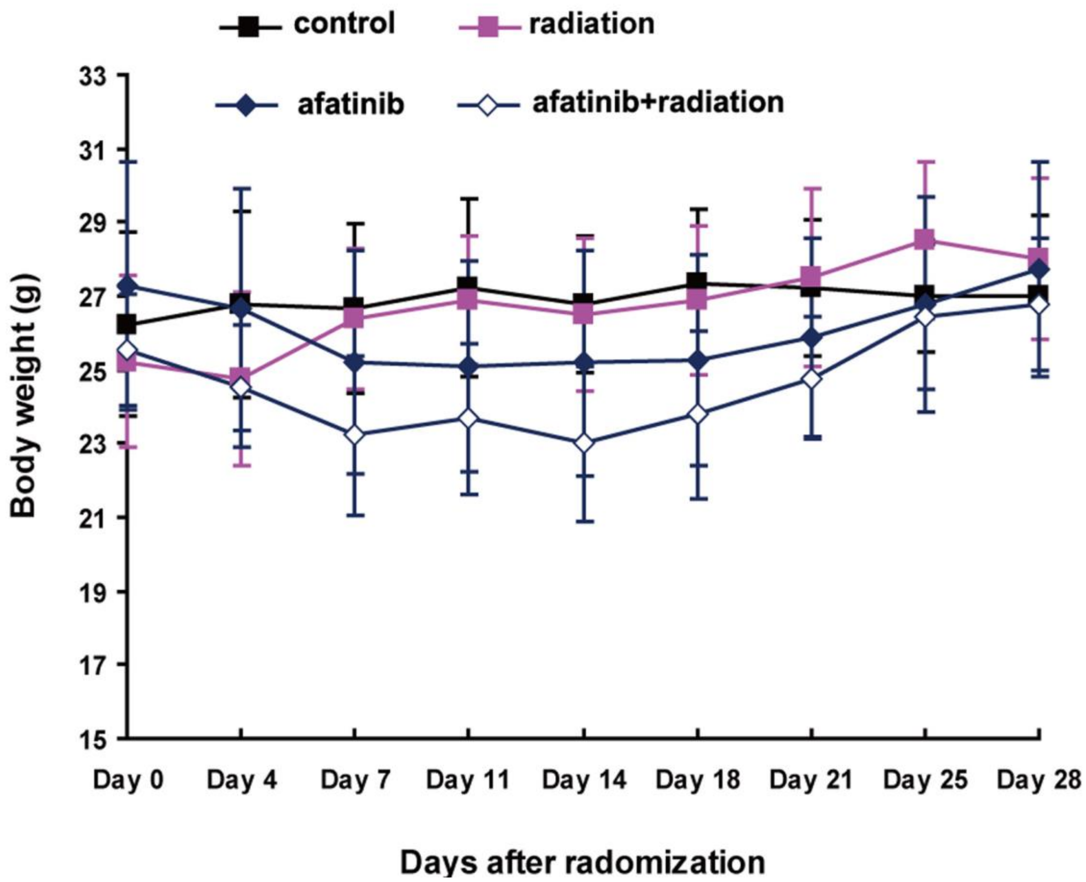

Supplementary figure S4: Diagram showing the body weight changes of mice for each group from day1 to day 28 post treatments. Error bars represent SD (n = 8 mice per group).
